# Nursing education for climate change: historical milestones and pedagogical axes for curricular integration

**DOI:** 10.1590/0034-7167-2025-0360

**Published:** 2026-08-21

**Authors:** Nádile Juliane Costa de Castro, Eudes José Braga, Alessandra Queiroz Conceição, Nyvia Cristina dos Santos Lima

**Affiliations:** I Universidade Federal do Pará. Belém, Pará, Brazil

**Keywords:** Climate Change, Nursing, Environmental Health, Nursing Education, Curriculum., Cambio Climático, Enfermaría, Salud Ambiental, Educación en Enfermería, Curriculum.

## Abstract

**Objectives:**

to analyze historical and conceptual milestones that support nursing education for addressing climate change, aligning with Sustainable Development Goals 3, 4, and 13, and to propose pedagogical axes for curricular integration.

**Methods:**

a qualitative, exploratory, documentary study, grounded in Freire’s critical perspective, which problematizes the relationships between global policies and local realities. Data collection took place between September 2024 and March 2025. Thematic analysis was used, guided by the problematization, dialogicity, and transformative praxis principles.

**Results:**

ten documents were analyzed. Five pedagogical axes emerged: historical evolution of the climate-health interface; critical knowledge for facing the climate reality; transformative training in climate and health; recognition and reduction of vulnerabilities; and collaborative and intersectoral action.

**Final Considerations:**

the axes provide a foundation for critical and emancipatory nursing education through Freire’s methodologies, promoting awareness of socio-environmental determinants of health and capacity building for advocacy in climate justice with vulnerable populations.

## RESUMEN

**Objetivos:**

analizar los hitos históricos y conceptuales que sustentan la formación en enfermería para abordar el cambio climático, en consonancia con los Objetivos de Desarrollo Sostenible 3, 4 y 13, y proponer ejes pedagógicos para la integración curricular.

**Métodos:**

estudio cualitativo, exploratorio y documental, basado en la perspectiva crítica de Freire, que problematiza las relaciones entre las políticas globales y las realidades locales. La recopilación de datos tuvo lugar entre septiembre de 2024 y marzo de 2025. Se utilizó un análisis temático, guiado por los principios de problematización, dialogicidad y praxis transformadora.

**Resultados:**

se analizaron diez documentos. Se identificaron cinco ejes pedagógicos: evolución histórica de la interfaz clima-salud; conocimiento crítico para afrontar la realidad climática; formación transformadora en clima y salud; reconocimiento y reducción de vulnerabilidades; y acción colaborativa e intersectorial.

**Consideraciones Finales:**

los ejes sientan las bases para una formación en enfermería crítica y emancipadora a través de metodologías de Freire, promoviendo la conciencia de los determinantes socioambientales de la salud y el desarrollo de capacidades para la incidencia en justicia climática con poblaciones vulnerables.

## INTRODUCTION

Climate change is one of the greatest global challenges of the 21^st^ century, directly and significantly affecting human health^(1)^. This impact is particularly exacerbated in populations residing in historically excluded territories, such as peripheral communities, *quilombola* communities, indigenous communities, *terreiro* (meeting places) communities, and riverside communities^(2)^ and from marginalized regions like the Amazon.

Paradoxically, although these populations contribute the least to greenhouse gas emissions, they are the ones that suffer the most from their adverse effects^(3)^. This disparity highlights the vulnerability of these groups^(1)^, placing them at the epicenter of climate impacts and demonstrating the urgency of incorporating the concept of climate justice as a guiding principle in the training of healthcare professionals^(3)^. The concept of climate justice recognizes that socioeconomic, cultural, and environmental inequalities amplify the effects of climate change, demanding interventions that consider the socioenvironmental context in which these populations are embedded^(4)^, and emerges as a central ethical imperative for nursing.

To this end, nursing education must promote practices anchored in critical, reflective, and socially engaged principles, incorporating the concept of climate justice as a central axis of professional practice^(4)^. In doing so, nurses become agents of social transformation, engaging in dialogue with health equity and possessing the skills to understand and address structural inequalities^(5,6)^, and contemporary environmental challenges, such as extreme weather events, *La Niña*, and *El Niño*.

Although some studies address planetary health, sustainable practices and environmental education^(5-8)^ in nursing education, it is necessary to advance in the interface with environmental health surveillance, education for sustainability and disaster risk management. The absence of content connected to local realities in the curriculum hinders the preparation of nurses capable of integrating interdisciplinary, intercultural approaches and epistemologies from the Global South.

Studies point to technical preparedness for environmental emergencies^(5)^, proposals that emphasize planetary health without problematizing structural inequalities^(1,3)^. However, there are gaps in critical pedagogical foundations that question the structures causing the climate crisis and systematic articulation between specific nursing skills and climate justice. This gap justifies the need for a proposal that transcends technical approaches or uncritically adapts models from the Global North to realities of the Global South^(5)^, compromising specific skills for risk management, disaster surveillance^(9)^ and popular health surveillance^(10)^. In this regard, pedagogical strategies aligned with curricular guidelines and the Sustainable Development Goals (SDGs) are essential.

This study is justified by its alignment with SDGs 3 (Good Health and Well-Being), 4 (Quality Education), and 13 (Climate Action), and with prioritized research areas such as black populations’ and traditional communities’ health, indigenous health, and health programs and policies. It is in accordance with the Brazilian National Curriculum Guidelines for Undergraduate Nursing Courses (In Portuguese, *Diretrizes Curriculares Nacionais do Curso de Graduação em Enfermagem* - DCN/Enf), which guide the training of critical, reflective, and socially committed nurses, capable of working in different sociocultural contexts and realities, considering the comprehensiveness of care actions and the socio-environmental determinants of health.

A comparative analysis of international initiatives reveals three predominant approaches - curricular enrichment, skills, and sustainability awareness -, which differ in their proposed structural transformation. This approach is based on Freire’s premise that truly transformative education must question the conditions that generate problems, not merely prepare people to live with their consequences.

Thus, this study seeks to answer the following question: what historical milestones and concepts should be considered in the training of nurses to address climate change, ensuring alignment with SDGs 3, 4 and 13 and DCN/Enf?

## OBJECTIVES

To analyze historical and conceptual milestones that support nursing education for addressing climate change, aligning with SDGs 3, 4, and 13, and to propose pedagogical axes for curricular integration.

## METHODS

### Ethical aspects

Since this analysis involved publicly accessible documents, approval from a Research Ethics Committee involving Human Beings was not required, in accordance with CNS Resolution 466/2012.

### Study design

This is a qualitative, exploratory, documentary study^(11)^, based on Freire’s assumptions of critical education^(12)^, in order to contribute to transformative pedagogical proposals, the construction of pedagogical axes aligned with SDGs and DCN/Enf, identifying silences and absences in institutional policies - a central element of Freire’s critique. The Consolidated criteria for Reporting Qualitative research tool was used for transparency and organization of the method section presentation^(13)^.

### Methodological procedures

Data sources were websites of the United Nations (UN), World Health Organization (WHO), Pan American Health Organization (PAHO), International Council of Nurses (ICN), Ministry of Environment and Climate Change (MMA), Ministry of Health (MoH), and Ministry of Education (MEC). The procedure for identifying the *corpus* on official websites included systematic navigation by relevance, using the search terms “climate change”, “environmental health”, “health education”, “nursing”, and “professional training”, applied individually and in Boolean combinations.

Inclusion criteria encompassed documents published between 2021 and 2024 to ensure timeliness and fundamental historical milestones, such as the Stockholm Declaration (1972), the Kyoto Protocol (1997), and the Paris Agreement (2015), due to their relevance for historical-conceptual contextualization, and availability of full text in Portuguese and English. Exclusion criteria included informative materials without normative or conceptual character. Both have an institutional and normative nature.

### Data collection and organization

Data collection was carried out between September 2024 and March 2025, with systematic extraction of the following variables: title; year; institution; and document type. For key concepts, those related to environmental health were defined, with definitions presented, professional skills, and suggested pedagogical and methodological approaches. Each document was read in its entirety by the two researchers independently, with individual note-taking and extraction of text segments.

### Data analysis

Triangulation of institutional perspectives was operationalized through a comparison among different orientations: international organizations (UN, WHO, PAHO); national policies (MMA, MoH, MEC); and professional guidelines (ICN). The analytical process prioritized not only conceptual convergences, but especially structural contradictions between different institutional perspectives. This comparison was carried out by recording, in a specific matrix, the contribution of each document to the construction of pedagogical topics and axes, allowing traceability and transparency in the analysis. Triangulation was not limited to the comparison of content, but involved a critical analysis of the different institutional rationalities, highlighting tensions and gaps.

Initial coding was performed segment by segment in the textual extracts, generating descriptive codes that were subsequently grouped by semantic similarity and theoretical relevance in an analysis spreadsheet, resulting in intermediate categories down to five main topics. To ensure analytical transparency, a document contribution matrix was constructed, which specifically recorded how each of the ten documents participated in the construction of each emerging topic. The final naming of pedagogical axes was based on the conceptual essence of each topic and its documentary foundation.

Code lists were subsequently compared, with systematic identification of convergences and divergences. Discrepancies in coding were resolved by consensus through return to the original document and reasoned discussion. The analytical sufficiency of the documentary *corpus* was achieved when the last three documents analyzed did not present new conceptual elements for the topics under construction. Data were systematized in Microsoft Word 365 for textual and categorical organization, and the figures were created in Canva Pro for visual presentation of results.

## RESULTS

Ten documents were identified, four from the UN, three from PAHO/WHO/MoH, one from MMA, one from MEC, and one from ICN. The results were organized into historical milestones, analyzed documents, key concepts, and pedagogical axes, in order to demonstrate the historical evolution and theoretical foundations, as observed in Chart 1.

**Chart 1:** Documentary *corpus,* Belém, Pará, Brazil, 2025

ORG.	DOCUMENT		ANO	TYPE
ONU	1	Stockholm Declaration	1972	Declaration
	2	Kyoto Protocol	1997	Treaty
	3	Paris Agreement	2015	Agreement
	4	Intergovernmental Panel on Climate Change	2023	Report
MoH/PAHO/WHO	5	Policy for Strengthening Equity-Oriented Health Sector Action on Climate Change and Health	2024	Institutional policy
	6	Climate Change for Health Professionals: A Pocket Book	2024	Technical guide
	7	G20 Health Document on Climate Change	2024	Ministerial statement
MMA	8	Nationally Determined Contribution of Brazil	2024	National policy
ICN	9	Guidelines: Essential competencies in disaster nursing	2022	Professional guidelines
MEC/CNE	10	Update of DCN/Enf 2024 - Process: 23001.000194/2016-12	2024	Regulatory resolution

*WHO - World Health Organization; MoH - Ministry of Health; PAHO - Pan American Health Organization; UN - United Nations; ICN - International Council of Nurses; MEC/CNE - Ministry of Education and National Education Council; MMA - Ministry of Environment and Climate Change; DCN/Enf - Brazilian National Curriculum Guidelines for Undergraduate Nursing Courses.*

After the selection and comprehensive analysis of the documents described in Chart 1, a systematic coding and categorization process was conducted, resulting in the identification of recurring conceptual patterns. Thematic analysis allowed for the emergence of five pedagogical axes, which synthesize the historical and conceptual milestones identified.

**Figure 1:**
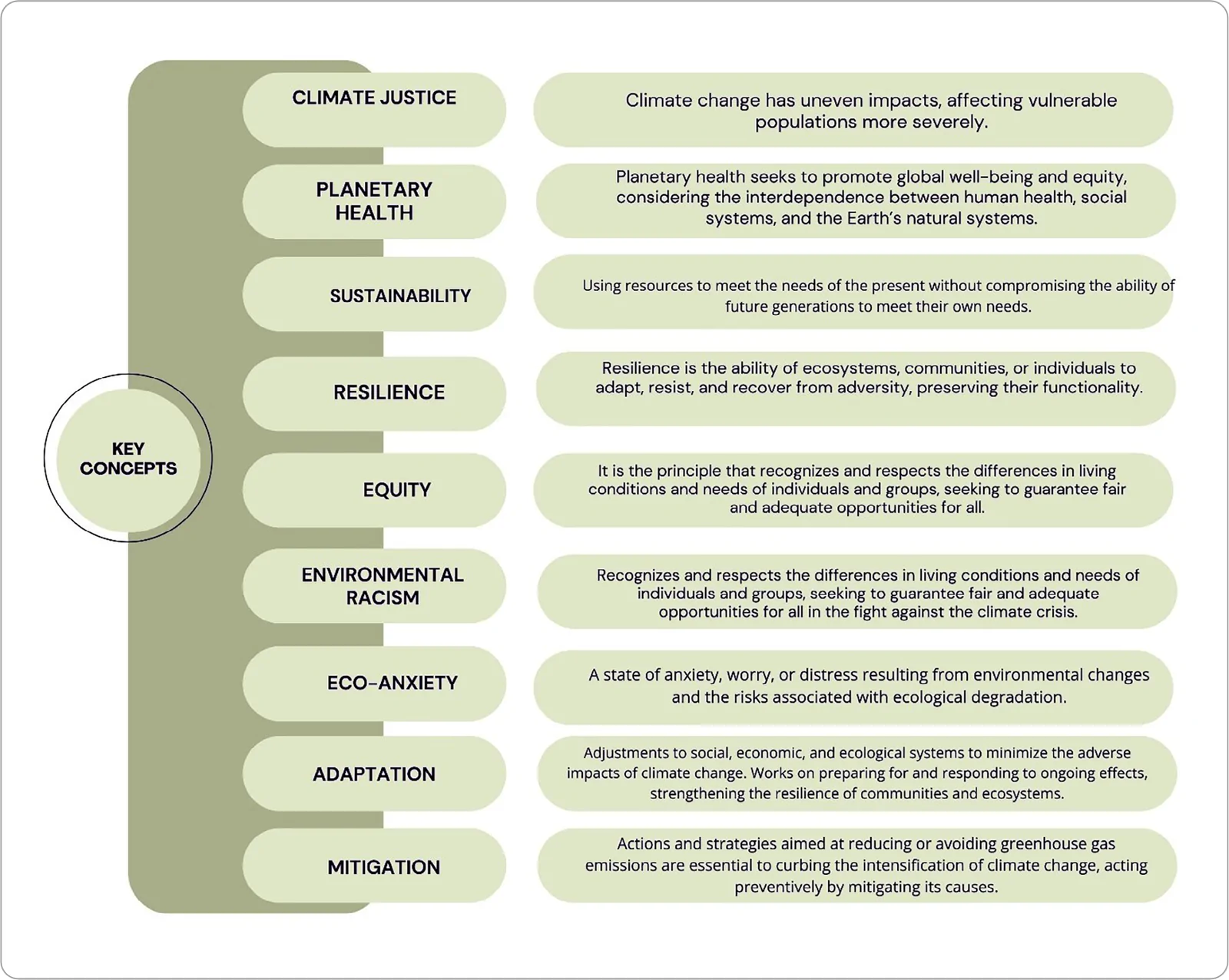
Key concepts, Belém, Pará, Brazil, 2025

From document analysis, key concepts emerged that support the integration of climate change into nursing education (Figure 1). Emerging key concepts reflect tensions between global perspectives and local needs. Climate justice appears in international documents.

**Figure 2:**
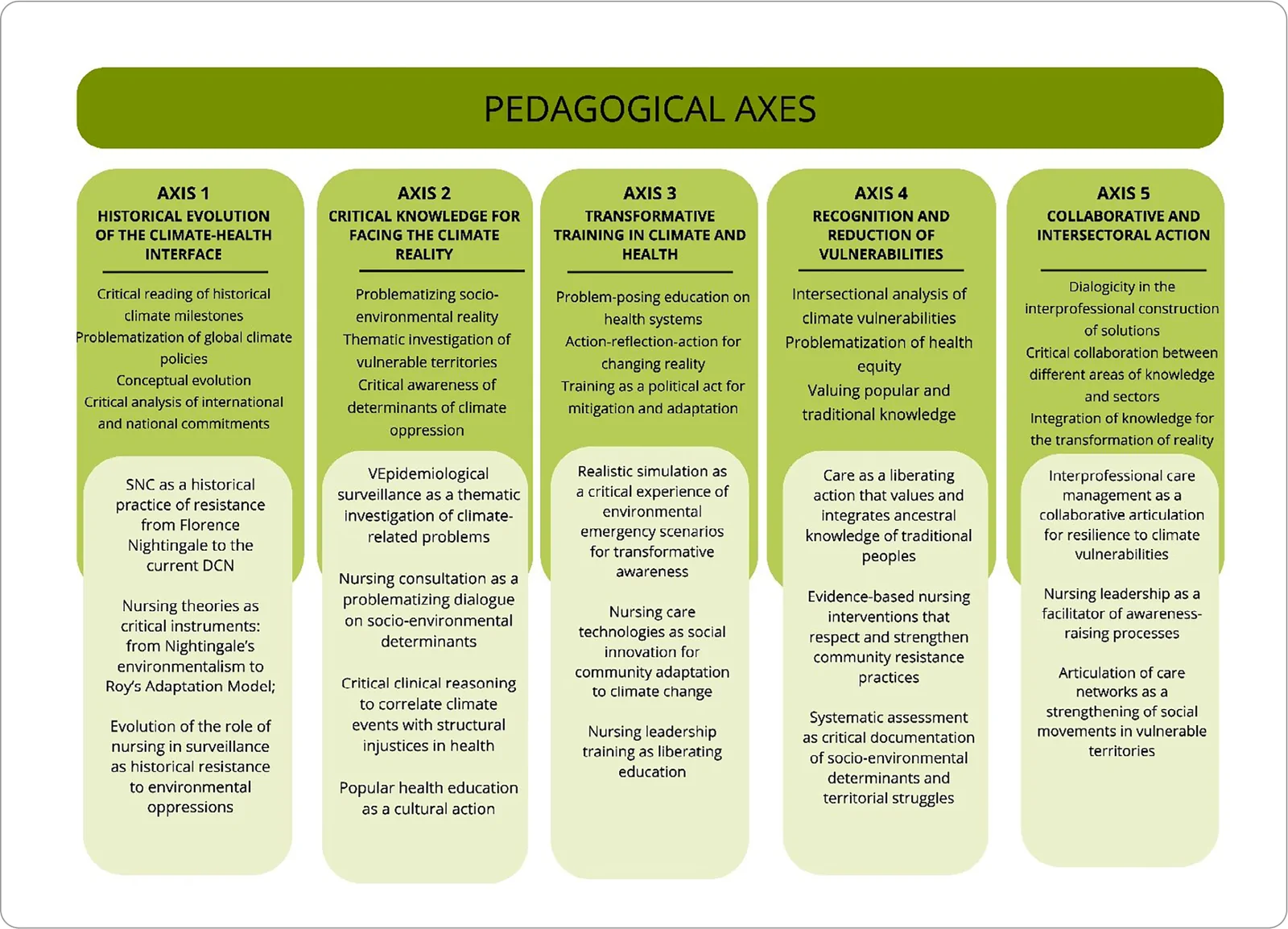
Pedagogical axes for curricular integration, Belém, Pará, Brazil, 2025

The document contribution matrix shows how each of the ten documents analyzed participated in the construction of pedagogical axes (Figure 2). Axis 1 was mainly based on global historical milestones (documents 1, 2, 3, 4, and 8), establishing the temporal evolution of the climate-health interface. Axes 2, 3, 4, and 5 presented contributions more distributed among the sources, with emphasis on contemporary technical-normative documents (5, 6, 7, 9, and 10), which specify critical-reflective capacities advocated for implementation strategies.

The key concepts identified (Figure 1) are structured around climate justice as a central articulating principle and do not constitute merely an additional concept, but a cross-cutting perspective that guides a critical understanding of all other elements.

The interrelationship among key concepts was structured into five complementary pedagogical axes (Figure 2), configuring an integrated proposal that operationalizes the inclusion of climate change in nursing education. Each axis articulates specific technical nursing competencies with critical-transformative perspectives, promoting training that transcends the uncritical reproduction of global guidelines.

The structured pedagogical axes are directly aligned with the graduate profile advocated by the following DCN/Enf 2024: critical and reflective training (axes 1 and 2); social and ethical commitment (axes 3 and 4); interprofessional work (axis 5); and continuing education (transversal to all five axes).

The analysis revealed a direct alignment of pedagogical axes with the specific goals of SDGs. Axis 1 connects with target 13.3 (education and awareness about climate change); axes 2 and 3 align with target 3.d (capacity for early warning and risk management in health); axis 4 relates to target 3.8 (universal health coverage and equity); and axis 5 connects with target 4.7 (education for sustainable development). The proposed training contributes to SDGs, but from a transformative perspective that questions not only “how” to achieve the goals, but “in whose service” they were formulated and whose voices were silenced in their creation.

## DISCUSSION

The analysis of historical milestones transcends chronological cataloging, revealing structural contradictions that permeate the formation of climate change^(14-16)^. This approach, grounded in Freire’s thought, allows us to understand that apparent advances in climate policies coexist with a deepening of socio-environmental inequalities^(1)^. A fundamental tension is evident between the universalism proclaimed in global policies and the particularities of territorial vulnerabilities. While the Stockholm Declaration establishes a universal right to a healthy environment^(5,14)^, contemporary documents recognize the unequal distribution of climate impacts, highlighting a contradiction between egalitarian discourse and stratified reality, which pedagogical axes should problematize^(17,18)^.

A paradigmatic dispute is identified between the hegemonic technocratic approach in WHO/PAHO documents and the critical-reflective perspective in DCN/Enf 2024. This tension reflects a correlation of forces between the biomedical model, which reduces climate change to risks to be managed, and the critical approach, which understands it as an expression of structural socioeconomic contradictions. This is also noted in the persistence of patterns of exclusion of the most affected populations from decision-making processes. This contradiction highlights the structural limitations of top-down solutions to problems that demand territorial protagonism and popular knowledge^(1,3,4)^.

Dialectical synthesis of these contradictions underpins the proposed pedagogical axes, which transcend the uncritical reproduction of global guidelines, pointing instead to action on the identified tensions^(19)^. They articulate a focus from the global to the territory (axes 1 and 4), integrating technical skills with criticality (axes 2 and 3), and promoting horizontality in interprofessional action (axis 5). It positions nursing as an agent of social transformation^(6,8,19)^, not just a recipient of global guidelines.

Axis 1 establishes a conceptual basis given the trajectory of climate policies from a public health perspective^(1,2)^, enabling students to have a non-linear understanding of the history of climate policies. Instead of a progressive evolution, it promotes an understanding of the contradictions that run from Stockholm to current commitments, developing critical awareness^(6,8)^ of structural determinants that perpetuate climate vulnerabilities^(1)^.

Axes 2 and 3 converge on the need to develop critical-reflective capacities in epidemiological surveillance, risk management, and health education, as evidenced in contemporary documents from the WHO, PAHO, and ICN^(9,19)^. Axes 4 and 5 reflect the ethical-political dimension of training, emphasizing climate justice and collaborative work as central elements for equitable action in contexts of socio-environmental inequalities^(3,4)^.

The implementation of pedagogical axes demands transcending the traditionally rigid disciplinary boundaries in nursing education^(5-8)^. Climate education^(6,7)^, by its systemic nature, requires interprofessional collaboration with environmental sciences (for understanding ecological determinants), public health (for population analysis of impacts), public policies (for normative contextualization), and social sciences (for addressing vulnerabilities). The proposed axes, although structured from a nursing perspective, were designed for interdisciplinary articulation. Axis 5 explicitly states this need^(8)^, while the other axes incorporate knowledge that transcends the specific field of nursing.

The key concepts identified in document analysis - climate justice, planetary health, equity, sustainability, and resilience - emerge as unifying elements among the proposed pedagogical axes^(15,16,20,21)^. Climate justice, in particular, positions itself as a cross-cutting concept that allows for reflection on inequalities, especially in Latin American countries marked by profound socioeconomic inequalities^(4)^. Pedagogical immersion in the territories of indigenous peoples^(22)^, *quilombola* communities, traditional communities, and other vulnerable populations^(23)^ contributes to a focus on collective care, reinforcing the connection with popular health surveillance practices^(10)^ and promoting curricular integration that develops sustainability attitudes^(24)^.

Another important milestone is the 2030 Agenda, which presents SDGs, reflects on local issues based on the targets in SDGs 3 and 13, and its interfaces with the Brazilian Health System (In Portuguese, *Sistema Único de Saúde* - SUS). In the context of SUS, these guidelines are fundamental for consolidating programs such as the Brazilian National Health Surveillance Program, the Brazilian National Program for Surveillance of Water Quality for Human Consumption (In Portuguese, *Programa Nacional de Vigilância da Qualidade da Água para Consumo Humano* - Vigiagua), and the Health Surveillance Program for Risks Associated with Disasters (In Portuguese, *Programa de Vigilância em Saúde dos Riscos Associados aos Desastres* - Vigidesastres)^(8,9)^. They have a direct impact on the protection of vulnerable populations, being important mechanisms to be explored. They contribute directly to existing public policies, such as Vigiagua and Vigidesastres^(24)^, which strengthen SUS response to environmental and health emergencies^(8,25,26)^.

This perspective is important for exploring SDGs that reproduce vertical patterns, with limited participation from populations most affected by climate change. This contradiction is evidenced in target 13.3, which emphasizes education and awareness, but is silent on the need to transform the economic structures^(1)^ that cause the climate crisis, limiting itself to technocratic solutions that preserve crisis-causing structures.

The alignment identified among the pedagogical axes and SDGs 3, 4 and 13, as well as with the skills advocated by DCN/Enf, proposes strengthening nurses’ comprehensive training^(14)^, and comprehensiveness of care actions considering the biological, psychological, social and environmental dimensions of the health-disease process^(14)^. By integrating climate change as a socio-environmental determinant, the capacity to understand and act upon factors that influence public health is strengthened, promoting equity and sustainability in healthcare^(16,26)^.

Regarding active methodologies, it is noted that they emerge as promising paths for the development of critical knowledge in interprofessional climate risk management^(6,15,16)^. These include strategies such as realistic simulation of environmental emergencies, case studies on specific territorial vulnerabilities, and outreach projects that integrate different healthcare professionals with community leaders.

The problematization of climate change as a generating topic that emerges from the reality of territories regarding sustainability, ethnic-racial relations, and environmental education throughout training enables a critical analysis of social and environmental inequalities^(20)^. This cross-cutting approach encourages the development of an integrative vision that values diversity and promotes equity, essential for practice in SUS. Experiences that allow the identification and analysis of climate vulnerabilities in different contexts can be carried out through case studies, extension projects^(20)^, and the integration of monitoring technologies, enabling strategic planning, risk assessment and the development of response protocols for climate emergencies.

The use of case studies on communities affected by environmental problems offers an opportunity to apply theoretical knowledge to real-world contexts^(6,15,16)^. Applying these discussions through pedagogical practice can be implemented to identify responses to socio-environmental vulnerabilities related to climate change^(20)^, contributing to the implementation of SDG 3 regarding health equity. This is because they favor addressing topics such as disaster preparedness and response with an interface to health risk management^(9)^.

On the other hand, discussing popular surveillance is central to this articulation^(10)^. Highlighting the role of nurses working together with community leaders in implementing climate justice to empower communities provides opportunities for dialogue about care from a territorial perspective^(22)^. It contributes to the promotion of socially responsible care through equitable strategies^(10)^, in the face of the different cultural^(19,22)^ and socioeconomic contexts of populations affected by climate change.

The implementation of pedagogical guidelines must consider specific Brazilian regional characteristics, especially in regions with significant social inequalities such as the Amazon and urban peripheries^(22)^. Strategies based on traditional knowledge and local resilience practices can be incorporated into the curriculum, allowing students to understand how to adapt global policies to the specific needs of each community^(22)^. Popular surveillance emerges as a central strategy^(10)^, providing opportunities for dialogue on territorialized care and socially responsible care promotion, through equitable strategies, in the face of the different cultural and socioeconomic contexts of populations affected by climate change^(19,22)^.

Regarding the ability to act at different levels of care and to promote health, addressing climate change and climate justice enriches training by providing a broader understanding of the factors that influence public health^(14)^. This is particularly relevant for working with vulnerable communities, which disproportionately suffer the effects of climate change^(14,16)^. Health education should integrate content that allows for an understanding of the interrelationship between environment and health, enabling professionals to develop adaptive and mitigating strategies through training that involves regional networks, service delivery, and technologies in Primary Health Care^(26)^.

The proposed pedagogical axes represent a significant curricular innovation by transcending the horizontal integration of content related to climate change in nursing education^(5,14)^. As shown in Figure 3, curricular implementation should occur through cross-curricular topics, contextualized supervised internships, and complementary activities that connect theory and practice^(5,26)^.

This integration requires a paradigm shift that overcomes knowledge fragmentation, favoring a systemic approach that prepares nurses to understand and act on the socio-environmental determinants of health in a critical and transformative manner^(20,21)^. The proposed cross-disciplinary approach aligns with Freire’s principles of problematizing reality, dialogicity, and praxis, providing a foundation for training that empowers professionals to question and transform the structural conditions that perpetuate climate vulnerabilities^(10,22)^.

The pedagogical strategies structured in Figure 3 highlight the need for methodological renewal that goes beyond traditional expository teaching, incorporating realistic simulation, community projects, and participatory action research as central elements of training^(19,23)^. For instance, the simulation of climate disaster scenarios allows for the development of critical-reflective and non-technical skills essential for emergency response, while territorial immersion projects enable an understanding of the specific vulnerabilities of different populations^(25,26)^.

They promote the development of critical thinking, collaborative decision-making, and the ability to adapt to complex and uncertain contexts-fundamental characteristics for nurses who will work in climate change scenarios^(15,16)^. Participatory action research, in particular, strengthens the connection between academia and the community, promoting the production of contextualized and culturally relevant knowledge^(20)^.

On the other hand, the implementation of the proposed pedagogical axes faces structural contradictions in the traditional nursing curriculum that must be explicitly problematized^(5)^. It reveals tensions between the overload of mandatory biomedical content and the emerging needs of climate education, highlighting that transformative integration demands profound paradigm shifts, not just the addition of new content^(5-8)^. This structural contradiction often results in a superficial approach to topics such as climate change, reduced to elective subjects or supplementary content, perpetuating fragmentation that the proposed pedagogical axes seek to overcome.

The integration of critical-reflective perspectives on climate change faces resistance from academic structures traditionally organized by biomedical specialties^(5)^. Professors trained in conventional paradigms may have limitations in addressing the intersectionality between health, environment, and social justice, requiring ongoing education processes that go beyond one-off training sessions on sustainability^(1,6)^. A dissociation is identified among institutional discourses that proclaim socio-environmental responsibility and educational practices that maintain traditional methodologies. Overcoming these curricular contradictions requires gradual and strategic implementation^(5,6,8)^ of the proposed pedagogical axes.

Implementation includes professor training, development of specific teaching materials, and the establishment of partnerships with healthcare services and community organizations^(8,14)^. Formative assessment, as suggested in Figure 2, requires a reformulation of traditional assessment processes, prioritizing reflection on reality for skills practice^(6)^. International experience suggests that curricular change processes in nursing are more effective when supported by strategies for faculty engagement^(5)^, development of external partnerships and adaptation of teaching resources.

On the other hand, elective courses or complementary activities, allowing for impact assessment and methodological refinement before full curricular integration^(6,16)^, are a promising path^(14,27)^. This approach facilitates faculty engagement and the identification of best practices adapted to the institutional context^(5,16)^. Furthermore, it is necessary to apply it in the awareness-raising (mapping and discussions), capacity building (teaching and pedagogical development)^(26)^, implementation (extension projects, elective course)^(6,12)^, and curricular integration (cross-cutting incorporation and systematization of good practices) phases^(5,27)^.

Future perspectives include the development of impact indicators to measure the effectiveness of climate change training in professional practice, as well as the establishment of collaborative networks among educational institutions for sharing experiences and resources^(7,26)^. The consolidation of this curricular proposal will contribute to positioning Brazilian nursing as a protagonist in responding to climate challenges, strengthening SUS and promoting health equity nationally and internationally^(19,25)^.

Recent studies demonstrate that climate and health education positively impacts the knowledge, confidence, and intention to act of nursing students^(28)^. International professional associations have developed specific recommendations for integrating climate mitigation strategies into nursing practice^(5,29)^, demonstrating that the proposed training aligns with global trends in the profession. The literature on natural disasters highlights specific challenges in nurses’ working conditions^(9)^, as demonstrated in recent research on working conditions after an earthquake in Turkey^(30)^, underlining the importance of adequate preparation during undergraduate studies.

### Study limitations

It is recognized that this study, conducted in the specific field of nursing, presents limitations regarding the interdisciplinary approach essential for climate education and methodological limitations regarding Freire’s intersubjectivity, which requires direct dialogue among subjects in knowledge construction. The limited documentary analysis may not fully capture perspectives from other areas such as environmental sciences, public policy, and public health. As such, it presents limitations due to temporal delimitation, the interpretative nature of the analysis, and the dynamism of policies and guidelines, which may be updated after data collection.

### Contributions to nursing, health, or public policy

This study contributes to the debate on a specific pedagogical design for integrating climate change into nursing education, and offers a theoretical and conceptual foundation that articulates socio-environmental determinants of health with critical professional training, empowering future nurses for territorially-based practice. In this regard, within the clinical-assistance context, the proposal enhances nurses’ clinical reasoning and decision-making by incorporating and contributing to the effective identification, analysis, and intervention in socio-environmental vulnerabilities. The proposed training points to practices for climate justice, health equity, and response to environmental emergencies, strengthening professionals’ advocacy and leadership capacity in defending SUS and promoting planetary health.

For education, the pedagogical axes, which transversally integrate environmental, social, and climate justice topics into the nursing curriculum, guide the development of critical, contextualized educational practices aligned with SDGs. The study identifies gaps and silences in institutional nursing education policies regarding climate education and planetary health, proposing an analytical framework that can guide future research on the effectiveness of curricula, the impact of pedagogical practices on nurses’ critical training, and the validity of the proposed axes.

## FINAL CONSIDERATIONS

This study identified fundamental historical and conceptual milestones that underpin the integration of climate change into nursing education, structuring five specific pedagogical axes. The key concepts identified - climate justice, planetary health, equity, sustainability, and resilience - position nursing as a profession committed to social and environmental transformation.

The alignment shown among the pedagogical axes and SDGs 3, 4, and 13, as well as with DCN/Enf, demonstrates that the proposal strengthens nurses’ comprehensive training. Its implementation, through active methodologies, realistic simulation, and community projects, mediated by Freire’s principles of dialogicity and praxis, empowers future professionals for critical and equitable action with vulnerable populations. The articulation between teaching, service, and community promotes contextualized training that values popular knowledge and territorial practices of resistance.

The proposed pedagogical axes are based on Freire’s premise that education should question oppressive structures, empowering nurses for professional advocacy and leadership in climate justice. This training transcends technical preparation for climate impacts, developing critical awareness of the structural determinants of socio-environmental vulnerabilities and the capacity for transformative action in historically marginalized territories. Therefore, research is suggested that investigates practical experiences of curricular implementation, and students’, professors’, and professionals’ perceptions regarding the proposed integration and empirical validity of the proposed pedagogical axes.

## Data Availability

The research data are not available.
