# Supplementary material for: Nursing education for climate change: historical milestones and pedagogical axes for curricular integration
Source: Rev Bras Enferm. 2026 Aug 21;79:e20250360. doi: 10.1590/0034-7167-2025-0360 (PMC13506212; doi:10.1590/0034-7167-2025-0360)
Supplement: Translation

# Translation: Formação em enfermagem para mudanças climáticas: marcos históricos e eixos pedagógicos para integração curricular

**DOI:** 10.1590/0034-7167-2025-0360pt

**Authors:** Nádile Juliane Costa de Castro, Eudes José Braga, Alessandra Queiroz Conceição, Nyvia Cristina dos Santos Lima

**Affiliations:** I Universidade Federal do Pará. Belém, Pará, Brasil

**Keywords:** Mudança Climática, Enfermagem, Saúde Ambiental, Educação em Enfermagem, Currículo.

## Abstract

**Objetivos:**

analisar marcos históricos e conceituais que subsidiem a formação em enfermagem para o enfrentamento das mudanças climáticas, alinhando-se aos Objetivos de Desenvolvimento Sustentável 3, 4 e 13, e propor eixos pedagógicos para integração curricular.

**Métodos:**

estudo qualitativo, exploratório, do tipo documental, fundamentado na perspectiva crítica freireana, que problematiza relações entre políticas globais e realidades locais. Realizou-se a coleta entre setembro 2024 e março de 2025. Utilizou-se análise temática orientada pelos princípios de problematização, dialogicidade e práxis transformadora.

**Resultados:**

analisaram-se dez documentos. Emergiram cinco eixos pedagógicos: evolução histórica da interface clima-saúde; saberes críticos para enfrentar a realidade climática; formação transformadora em clima e saúde; reconhecimento e redução de vulnerabilidades; e atuação colaborativa e intersetorial.

**Considerações Finais:**

os eixos fundamentam formação crítica e emancipatória em enfermagem através de metodologias freireanas, promovendo conscientização sobre determinantes socioambientais da saúde e capacitação para advocacia em justiça climática junto às populações vulnerabilizadas.

## INTRODUÇÃO

As mudanças climáticas constituem um dos maiores desafios globais do século XXI, afetando direta e significativamente a saúde humana^(1)^. Esse impacto é particularmente exacerbado em populações que residem em territórios historicamente excluídos, como comunidades periféricas, quilombolas, indígenas, de terreiros, ribeirinhas^(2)^ e de regiões marginalizadas como da Amazônia.

Paradoxalmente, embora essas populações sejam as que menos contribuem para as emissões de gases de efeito estufa, são as que mais sofrem com seus efeitos adversos^(3)^. Essa disparidade evidencia a vulnerabilidade desses grupos^(1)^, posicionando-os no epicentro dos impactos climáticos e demonstrando a urgência de incorporar o conceito de justiça climática como princípio orientador na formação de profissionais de saúde^(3)^. O conceito de justiça climática reconhece que as desigualdades socioeconômicas, culturais e ambientais amplificam os efeitos das mudanças climáticas, demandando intervenções que considerem o contexto socioambiental em que essas populações estão inseridas^(4)^, e emerge como imperativo ético central para a enfermagem.

Para isso, a formação em enfermagem deve promover práticas ancoradas em princípios críticos, reflexivos e socialmente engajados, incorporando o conceito de justiça climática como eixo central da prática profissional^(4)^. Ao fazê-lo, enfermeiros se tornam agentes de transformação social, em dialogicidade com a equidade em saúde e com aptidão para compreender e enfrentar as desigualdades estruturais^(5,6)^ e os desafios ambientais contemporâneos, como eventos climáticos extremos, *La Niña* e *El Niño*.

Embora alguns estudos abordem saúde planetária, práticas sustentáveis e educação ambiental^(5-8)^ na formação em enfermagem, é necessário avançar na interface com vigilância em saúde ambiental, educação para sustentabilidade e gestão de riscos de desastres. A ausência de conteúdos articulados com realidades locais no currículo dificulta a preparação de enfermeiros capazes de integrar abordagens interdisciplinares, interculturais e epistemologias do Sul Global.

Estudos apontam preparação técnica para emergências ambientais^(5)^, propostas que enfatizam saúde planetária sem problematizar desigualdades estruturais^(1,3)^. Entretanto, há lacunas sobre fundamentação pedagógica crítica que questione estruturas causadoras da crise climática e articulação sistemática entre habilidades específicas da enfermagem e justiça climática. Esta lacuna justifica a necessidade de proposta que transcenda abordagens técnicas ou adapte acriticamente modelos do Norte Global para realidades do Sul Global^(5)^, comprometendo habilidades específicas para gestão de riscos, vigilância de desastres^(9)^ e vigilância popular em saúde^(10)^. Neste sentido, é essencial estratégias pedagógicas alinhadas às diretrizes curriculares e aos Objetivos de Desenvolvimento Sustentável (ODS).

Este estudo é justificado por se alinhar aos ODS 3 (Saúde e Bem-Estar), 4 (Educação de qualidade) e 13 (Ação contra a Mudança Global do Clima), e às linhas de pesquisa priorizadas, como saúde da população negra e das comunidades tradicionais, saúde indígena, e programas e políticas em saúde. Está em conformidade com as Diretrizes Curriculares Nacionais do Curso de Graduação em Enfermagem (DCN/Enf), que orientam a formação de enfermeiros críticos, reflexivos e socialmente comprometidos, e aptos a atuar em diferentes contextos e realidades socioculturais, considerando a integralidade das ações de cuidado e os determinantes socioambientais da saúde.

A análise comparativa de iniciativas internacionais evidencia três abordagens predominantes - adição curricular, habilidades e conscientização sobre sustentabilidade -, diferenciando-se ao propor transformação estrutural. Esta abordagem é fundamentada na premissa freireana de que educação verdadeiramente transformadora deve questionar as condições que geram os problemas, não apenas preparar para conviver com suas consequências.

Assim, este estudo busca responder à seguinte questão: quais marcos históricos e conceitos devem ser considerados na formação de enfermeiros para o enfrentamento das mudanças climáticas, garantindo alinhamento aos ODS 3, 4 e 13 e às DCN/Enf?

## OBJETIVOS

Analisar marcos históricos e conceituais que subsidiem a formação em enfermagem para o enfrentamento das mudanças climáticas, alinhando-se aos ODS 3, 4 e 13, e propor eixos pedagógicos para integração curricular.

## MÉTODOS

### Aspectos éticos

Por se tratar de análise de documentos de acesso público, não houve necessidade de aprovação por Comitê de Ética em Pesquisa com Seres Humanos, conforme Resolução CNS n^o^ 466/2012.

### Tipos de estudo

Estudo qualitativo, exploratório, do tipo documental^(11)^, fundamentado nos pressupostos freireanos de educação crítica^(12)^, a fim de colaborar com proposições pedagógicas transformadoras, construção de eixos pedagógicos alinhados aos ODS e DCN/Enf, identificando silêncios e ausências nas políticas institucionais - elemento central da crítica freireana. Foi utilizado o *COnsolidated criteria for REporting Qualitative research* para fins de transparência e organização da apresentação da seção método^(13)^.

### Procedimentos metodológicos

As fontes de dados foram sítios eletrônicos da Organização das Nações Unidas (ONU), Organização Mundial da Saúde (OMS), Organização Pan-Americana da Saúde (OPAS), do *International Council of Nurses* (ICN), Ministério do Meio Ambiente e Mudança do Clima (MMA), Ministério da Saúde (MS) e Ministério da Educação (MEC). O procedimento de identificação do *corpus* em sítios oficiais incluiu navegação sistemática por relevância, utilizando-se os termos de busca “mudanças climáticas”, “saúde ambiental”, “educação em saúde”, “enfermagem” e “formação profissional”, aplicados individualmente e em combinações booleanas.

Os critérios de inclusão abrangeram documentos publicados entre 2021 e 2024 para garantir atualidade e marcos históricos fundamentais, como a Declaração de Estocolmo (1972), o Protocolo de Kyoto (1997) e o Acordo de Paris (2015), devido à sua relevância para contextualização histórico-conceitual, e disponibilidade de texto completo e idiomas português e inglês. Os critérios de exclusão compreenderam materiais informativos sem caráter normativo ou conceitual. Ambos possuem natureza institucional e normativa.

### Coleta e organização dos dados

A coleta de dados foi realizada entre setembro de 2024 e março de 2025, com extração sistemática das seguintes variáveis: título; ano; instituição; e tipo de documento. Para conceitos-chave, definiram-se os relacionados à saúde ambiental com definições apresentadas, habilidades profissionais, abordagens pedagógicas e metodológicas sugeridas. Cada documento foi lido integralmente pelos dois pesquisadores independentemente, com fichamento individual e extração de segmentos textuais.

### Análise dos dados

A triangulação de perspectivas institucionais foi operacionalizada através do confronto entre diferentes orientações: organismos internacionais (ONU, OMS, OPAS); políticas nacionais (MMA, MS, MEC); e diretrizes profissionais (ICN). O processo analítico priorizou não apenas convergências conceituais, mas especialmente contradições estruturais entre diferentes perspectivas institucionais. Esse confronto foi realizado registrando, em matriz específica, a contribuição de cada documento para a construção dos temas e eixos pedagógicos, permitindo rastreabilidade e transparência na análise. A triangulação não se limitou à comparação de conteúdos, mas envolveu uma análise crítica das diferentes racionalidades institucionais, evidenciando tensões e lacunas.

A codificação inicial foi realizada por segmento nos extratos textuais, gerando códigos descritivos que foram posteriormente agrupados por similaridade semântica e relevância teórica em planilha de análise, originando categorias intermediárias até cinco temas principais. Para assegurar transparência analítica, foi construída matriz de contribuição documental, que registrou especificamente como cada um dos dez documentos participou na construção de cada tema emergente. A nomeação final dos eixos pedagógicos foi embasada na essência conceitual de cada tema e sua fundamentação documental.

As listas de códigos foram posteriormente comparadas, com identificação sistemática de convergências e divergências. As discrepâncias na codificação foram resolvidas por consenso mediante retorno ao documento original e discussão fundamentada. A suficiência analítica do *corpus* documental foi alcançada quando os últimos três documentos analisados não apresentaram elementos conceituais inéditos para os temas em construção. Os dados foram sistematizados no *Microsoft Word 365* para organização textual e categórica, e as figuras foram elaboradas no *Canva Pro* para apresentação visual dos resultados.

## RESULTADOS

Foram identificados dez documentos, sendo que quatro são da ONU, três, da OPAS/OMS/MS, um, do MMA, um, do MEC, e um, do ICN. Os resultados foram organizados em marcos históricos, documentos analisados, conceitos-chave e eixos pedagógicos, a fim de demonstrar a evolução histórica e os fundamentos teóricos, como observado no Quadro 1.

**Quadro 1:** Corpus **documental, Belém, Pará, Brasil, 2025**

ORG.	DOCUMENTO		ANO	TIPO
ONU	1	Declaração de Estocolmo	1972	Declaração
	2	Protocolo de Kyoto	1997	Tratado
	3	Acordo de Paris	2015	Acordo
	4	Painel Intergovernamental sobre Mudanças Climáticas	2023	Relatório
MS/OPAS/OMS	5	Política sobre saúde, mudanças climáticas e equidade	2024	Política institucional
	6	Guia sobre Mudanças Climáticas para Profissionais da Saúde	2024	Guia técnico
	7	Documento do G20 Saúde sobre Mudanças Climáticas	2024	Declaração ministerial
MMA	8	Contribuição Nacionalmente Determinada do Brasil	2024	Política nacional
ICN	9	Diretrizes: Competências essenciais em enfermagem de desastres	2022	Diretrizes profissionais
MEC/CNE	10	Atualização das DCN/Enf 2024- Processo: 23001.000194/2016-12	2024	Resolução normativa

*OMS - Organização Mundial da Saúde; MS - Ministério da Saúde; OPAS - Organização Panamericana da Saúde; ONU - Organização das Nações Unidas; ICN - International Council of Nurses; MEC/CNE - Ministério da Educação e Conselho Nacional de Educação; MMA - Ministério do Meio Ambiente e Mudança do Clima; DCN/Enf - Diretrizes Curriculares Nacionais do Curso de Graduação em Enfermagem.*

Após a seleção e análise integral dos documentos descritos no Quadro 1, foi conduzido processo sistemático de codificação e categorização, que resultou na identificação de padrões conceituais recorrentes. A análise temática permitiu a emergência de cinco eixos pedagógicos, que sintetizam os marcos históricos e conceituais identificados.

Da análise documental, emergiram conceitos-chave fundamentais que sustentam a integração das mudanças climáticas na formação em enfermagem (Figura 1). Os conceitos-chave emergentes refletem tensões entre perspectivas globais e necessidades locais. Justiça climática aparece nos documentos internacionais.

**Figura 1:** Conceitos-chave, Belém, Pará, Brasil, 2025

A matriz de contribuição documental evidencia como cada um dos dez documentos analisados participou na construção dos eixos pedagógicos (Figura 2). O eixo 1 foi fundamentado principalmente pelos marcos históricos globais (documentos 1, 2, 3, 4 e 8), estabelecendo a evolução temporal da interface clima-saúde. Os eixos 2, 3, 4 e 5 apresentaram contribuições mais distribuídas entre as fontes, com destaque para os documentos técnico-normativos contemporâneos (5, 6, 7, 9 e 10), que especificam capacidades crítico-reflexivas preconizadas para estratégias de implementação.

**Figura 2:** Eixos pedagógicos para integração curricular, Belém, Pará, Brasil, 2025

Os conceitos-chave identificados (Figura 1) se estruturam em torno da justiça climática como princípio articulador central e não constitui apenas um conceito adicional, mas perspectiva transversal que orienta compreensão crítica de todos os demais elementos.

A inter-relação entre os conceitos-chave se estruturou em cinco eixos pedagógicos complementares (Figura 2), configurando proposta integrada que operacionaliza a inserção das mudanças climáticas na formação em enfermagem. Cada eixo articula competências técnicas específicas da enfermagem com perspectivas crítico-transformadoras, promovendo formação que transcende a reprodução acrítica de diretrizes globais.

Os eixos pedagógicos estruturados se alinham diretamente ao perfil do egresso preconizado pelas seguintes DCN/Enf 2024: formação crítica e reflexiva (eixos 1 e 2); compromisso social e ético (eixos 3 e 4); trabalho interprofissional (eixo 5); e educação permanente (transversal aos cinco eixos).

A análise evidenciou alinhamento direto dos eixos pedagógicos com as metas específicas dos ODS. O eixo 1 se conecta com a meta 13.3 (educação e conscientização sobre mudanças climáticas); os eixos 2 e 3 se alinham à meta 3.d (capacidade para alerta precoce e gestão de riscos em saúde); o eixo 4 se relaciona com a meta 3.8 (cobertura universal de saúde e equidade); e o eixo 5 se conecta com a meta 4.7 (educação para desenvolvimento sustentável). A formação proposta contribui para os ODS, mas a partir de uma perspectiva transformadora que questiona não apenas “como”alcançar os objetivos, mas “a serviço de quem” eles foram formulados e cujas vozes foram silenciadas em sua criação.

## DISCUSSÃO

A análise dos marcos históricos transcende catalogação cronológica, revelando contradições estruturais que atravessam a formação para mudanças climáticas^(14-16)^. Essa abordagem, fundamentada na abordagem freireana, permite compreender que avanços aparentes nas políticas climáticas coexistem com aprofundamento de desigualdades socioambientais^(1)^. Evidencia-se tensão fundamental entre universalismo proclamado nas políticas globais e particularidades das vulnerabilidades territoriais. Enquanto a Declaração de Estocolmo estabelece direito universal ao meio ambiente saudável^(5,14)^, documentos contemporâneos reconhecem distribuição desigual dos impactos climáticos, evidenciando contradição entre discurso igualitário e realidade estratificada, que os eixos pedagógicos devem problematizar^(17,18)^.

Identifica-se disputa paradigmática entre abordagem tecnicista hegemônica em documentos da OMS/OPAS e a perspectiva crítico-reflexiva nas DCN/Enf 2024. Essa tensão reflete correlação de forças entre modelo biomédico, que reduz mudanças climáticas a riscos a serem gerenciados, e abordagem crítica, que as compreende como expressão de contradições socioeconômicas estruturais. Isso também é notado na persistência de padrões de exclusão de populações mais afetadas dos processos decisórios. Essa contradição evidencia limitações estruturais de soluções verticais para problemas que demandam protagonismo territorial e saberes populares^(1,3,4)^.

A síntese dialética dessas contradições fundamenta os eixos pedagógicos propostos, que transcendem reprodução acrítica de diretrizes globais, apontando atuação sobre as tensões identificadas^(19)^. Articulam foco do global ao território (eixos 1 e 4), integrando habilidades técnicas com criticidade (eixos 2 e 3), e promovendo horizontalidade na ação interprofissional (eixo 5). Posiciona a enfermagem como agente de transformação social^(6,8,19)^, não apenas receptora de diretrizes globais.

O eixo 1 estabelece base conceitual dada trajetória das políticas climáticas desde perspectiva de saúde pública^(1,2)^, operacionalizando que estudantes tenham compreensão não linear da história das políticas climáticas. Em vez de evolução progressiva, promove compreensão das contradições que atravessam desde Estocolmo até os compromissos atuais, desenvolvendo consciência crítica^(6,8)^ sobre determinantes estruturais que perpetuam vulnerabilidades climáticas^(1)^.

Os eixos 2 e 3 convergem na necessidade de desenvolver capacidades crítico-refletivas em vigilância epidemiológica, gestão de riscos e educação em saúde, conforme evidenciado nos documentos contemporâneos da OMS, OPAS e ICN^(9,19)^. Já os eixos 4 e 5 refletem a dimensão ético-política da formação, enfatizando justiça climática e trabalho colaborativo como elementos centrais para atuação equitativa em contextos de desigualdades socioambientais^(3,4)^.

A implementação dos eixos pedagógicos demanda transcendência das fronteiras disciplinares tradicionalmente rígidas na formação em enfermagem^(5-8)^. A educação climática^(6,7)^, por sua natureza sistêmica, exige colaboração interprofissional com ciências ambientais (para compreensão de determinantes ecológicos), saúde pública (para análise populacional de impactos), políticas públicas (para contextualização normativa) e ciências sociais (para abordagem de vulnerabilidades). Os eixos propostos, embora estruturados desde a perspectiva da enfermagem, foram concebidos para articulação interdisciplinar. O eixo 5 explicita essa necessidade^(8)^, enquanto os demais eixos incorporam saberes que transcendem o campo específico da enfermagem.

Os conceitos-chave identificados na análise documental - justiça climática, saúde planetária, equidade, sustentabilidade e resiliência - emergem como elementos articuladores entre os eixos pedagógicos propostos^(15,16,20,21)^. A justiça climática, particularmente, posiciona-se como conceito transversal que permite reflexão sobre desigualdades, especialmente em países latino-americanos marcados por profundas desigualdades socioeconômicas^(4)^. A imersão pedagógica nos territórios de povos indígenas^(22)^, quilombolas, comunidades tradicionais e demais populações vulnerabilizadas^(23)^ colabora para o foco no cuidado coletivo, reforçando a articulação com práticas de vigilância popular em saúde^(10)^ e promovendo integração curricular que desenvolva atitudes de sustentabilidade^(24)^.

Outro marco importante é a Agenda 2030, que apresenta os ODS, a reflexão de questões locais a partir das metas nos ODS 3 e 13, e as interfaces com o Sistema Único de Saúde (SUS). No contexto do SUS, essas orientações são fundamentais para consolidar programas como o Programa Nacional de Vigilância em Saúde, Programa Nacional de Vigilância da Qualidade da Água para Consumo Humano (Vigiagua) e Programa de Vigilância em Saúde dos Riscos Associados aos Desastres (Vigidesastres)^(8,9)^. Eles têm impacto direto na proteção de populações vulnerabilizadas, sendo importantes mecanismos a serem explorados. Contribuem diretamente para políticas públicas já existentes, como o Vigiagua e o Vigidesastres^(24)^, que fortalecem a resposta do SUS às emergências ambientais e sanitárias^(8,25,26)^.

Essa perspectiva é importante para explorar os ODS que reproduzem padrões verticais, com limitada participação de populações mais afetadas pelas mudanças climáticas. Essa contradição é evidenciada na meta 13.3, que enfatiza educação e conscientização, mas silencia sobre a necessidade de transformação das estruturas econômicas^(1)^ que causam a crise climática, limitada às soluções tecnocráticas que preservam estruturas causadoras da crise.

O alinhamento identificado entre os eixos pedagógicos e os ODS 3, 4 e 13, bem como com as habilidades preconizadas pelas DCN/Enf, propõe fortalecimento da formação integral do enfermeiro^(14)^, e integralidade das ações de cuidado considerando dimensões biológica, psicológica, social e ambiental do processo de saúde-doença^(14)^. Ao integrar mudanças climáticas como determinante socioambiental, fortalece-se a capacidade de compreender e atuar sobre fatores que influenciam a saúde coletiva, promovendo equidade e sustentabilidade na atenção à saúde^(16,26)^.

Em relação às metodologias ativas, refere-se que emergem como caminhos promissores para desenvolvimento de saberes críticos em gestão interprofissional de riscos climáticos^(6,15,16)^. Estas incluem estratégias como simulação realística de emergências ambientais, estudos de caso sobre vulnerabilidades territoriais específicas e projetos de extensão que integrem diferentes profissionais de saúde com lideranças comunitárias.

A problematização das mudanças climáticas como tema gerador que emerja da realidade dos territórios sobre sustentabilidade, relações étnico-raciais e educação ambiental ao longo da formação possibilita análise crítica das desigualdades sociais e ambientais^(20)^. Essa transversalidade estimula desenvolvimento de visão integradora que valorize diversidade e promova equidade, essenciais para prática no SUS. Experiências que permitam identificar e analisar vulnerabilidades climáticas em diferentes contextos podem ser realizadas através de estudos de caso, projetos de extensão^(20)^ e integração de tecnologias de monitoramento, possibilitando planejamento estratégico, avaliação de riscos e elaboração de protocolos de atendimento para emergências climáticas.

O uso dos estudos de caso sobre comunidades afetadas por problemas ambientais oferece oportunidade de aplicar conhecimentos teóricos a contextos reais^(6,15,16)^. A aplicação dessas discussões por meio prática pedagógica pode ser implementada para identificar respostas às vulnerabilidades socioambientais relacionadas às mudanças climáticas^(20)^, contribuindo para implementar a ODS 3 referente à equidade em saúde. Isso ocorre porque favorecem abordagem de temas como preparação e resposta a desastres com interface à gestão de riscos em saúde^(9)^.

Por outro lado, discutir vigilância popular é central nessa articulação^(10)^. Sinalizar a relação da atuação de enfermeiros em conjunto com líderes comunitários na implementação da justiça climática para empoderamento das comunidades oportuniza diálogos sobre cuidados a partir dos territórios^(22)^. Colabora para promoção de cuidado socialmente responsável através de estratégias equitativas^(10)^, diante dos diferentes contextos culturais^(19,22)^ e socioeconômicos das populações afetadas pelas mudanças climáticas.

A implementação dos eixos pedagógicos deve considerar especificidades regionais brasileiras, especialmente em regiões com grandes desigualdades sociais como Amazônia e periferias urbanas^(22)^. Estratégias baseadas em saberes tradicionais e práticas de resiliência local podem ser incorporadas ao currículo, permitindo aos estudantes compreender como adaptar políticas globais às necessidades específicas de cada comunidade^(22)^. A vigilância popular emerge como estratégia central^(10)^, oportunizando diálogos sobre cuidados territorializados e promoção de cuidado socialmente responsável, através de estratégias equitativas, diante dos diferentes contextos culturais e socioeconômicos das populações afetadas pelas mudanças climáticas^(19,22)^.

No que tange à capacidade de atuação em diferentes níveis de atenção e à promoção da saúde, a abordagem das mudanças climáticas e da justiça climática enriquece a formação ao proporcionar compreensão ampliada dos fatores que influenciam a saúde coletiva^(14)^. Isso é particularmente relevante para atuação junto a comunidades vulnerabilizadas, que sofrem desproporcionalmente os efeitos das mudanças climáticas^(14,16)^. A formação em saúde deve integrar conteúdos que permitam compreender a interrelação entre meio ambiente e saúde, capacitando profissionais para desenvolver estratégias adaptativas e mitigadoras por meio de capacitações que envolvam redes regionais, prestação de serviços e tecnologias na Atenção Primária à Saúde^(26)^.

A proposta de eixos pedagógicos representa inovação curricular significativa ao transcender integração horizontal dos conteúdos relacionados às mudanças climáticas na formação em enfermagem^(5,14)^. Conforme evidenciado na Figura 3, a operacionalização curricular deve ocorrer através de transversalidades, estágios supervisionados contextualizados e atividades complementares que conectem teoria e prática^(5,26)^.

Essa integração exige mudança paradigmática que supere a fragmentação do conhecimento, favorecendo abordagem sistêmica que prepare enfermeiros para compreender e atuar sobre determinantes socioambientais da saúde de forma crítica e transformadora^(20,21)^. A transversalidade proposta se alinha aos princípios freireanos de problematização da realidade, dialogicidade e práxis, fundamentando formação que capacite profissionais para questionar e transformar as condições estruturais que perpetuam vulnerabilidades climáticas^(10,22)^.

As estratégias pedagógicas estruturadas na Figura 3 evidenciam necessidade de renovação metodológica que vá além do ensino tradicional expositivo, incorporando simulação realística, projetos comunitários e pesquisa-ação participativa como elementos centrais da formação^(19,23)^. A simulação de cenários de desastres climáticos, por exemplo, permite desenvolvimento de capacidades crítico-refletivas e não técnicas essenciais para atuação em emergências, enquanto projetos de imersão territorial possibilitam compreensão das vulnerabilidades específicas de diferentes populações^(25,26)^.

Favorecem desenvolvimento de pensamento crítico, tomada de decisão colaborativa e capacidade de adaptação a contextos complexos e incertos, características fundamentais para enfermeiros que atuarão em cenários de mudanças climáticas^(15,16)^. A pesquisa-ação participativa, particularmente, fortalece articulação entre academia e comunidade, promovendo produção de conhecimento contextualizado e culturalmente relevante^(20)^.

Por outro lado, a implementação dos eixos pedagógicos propostos enfrenta contradições estruturais do currículo tradicional de enfermagem que devem ser explicitamente problematizadas^(5)^. Revela tensões entre sobrecarga de conteúdos biomédicos obrigatórios e necessidades emergentes de educação climática, evidenciando que integração transformadora demanda mudanças paradigmáticas profundas, não apenas adição de novos conteúdos^(5-8)^. Essa contradição estrutural resulta frequentemente em abordagem superficial de temáticas como mudanças climáticas, reduzidas a disciplinas optativas ou conteúdos complementares, perpetuando fragmentação que os eixos pedagógicos propostos buscam superar.

A integração de perspectivas crítico-reflexivas sobre mudanças climáticas enfrenta resistências de estruturas acadêmicas tradicionalmente organizadas por especialidades biomédicas^(5)^. Docentes formados em paradigmas convencionais podem apresentar limitações para abordar interseccionalidade entre saúde, ambiente e justiça social, demandando processos de educação permanente que transcendam capacitações pontuais sobre sustentabilidade^(1,6)^. Identifica-se dissociação entre discursos institucionais que proclamam responsabilidade socioambiental e práticas educacionais que mantêm metodologias tradicionais. A superação dessas contradições curriculares exige implementação gradual e estratégica^(5,6,8)^ dos eixos pedagógicos propostos.

A implementação inclui capacitação docente, desenvolvimento de materiais didáticos específicos e estabelecimento de parcerias com serviços de saúde e organizações comunitárias^(8,14)^. A avaliação formativa, sugerida na Figura 2, exige reformulação dos processos avaliativos tradicionais, priorizando reflexão da realidade para práticas de habilidades^(6)^. A experiência internacional sugere que processos de mudança curricular em enfermagem são mais efetivos quando sustentados por estratégias de engajamento docente^(5)^, desenvolvimento de parcerias externas e adequação de recursos pedagógicos.

Por outro lado, disciplinas optativas ou atividades complementares, permitindo avaliação de impacto e refinamento metodológico antes da integração^(6,16)^ curricular completa, são um caminho promissor^(14,27)^. Essa abordagem facilita adesão de docente e identificação de melhores práticas adaptadas ao contexto institucional^(5,16)^. Além disso, é necessária a aplicação nas fases de sensibilização (mapeamento e discussões), capacitação (docente e de desenvolvimento pedagógico)^(26)^, implementação (projetos de extensão, disciplina optativa)^(6,12)^ e integração curricular (incorporação transversal e sistematização de boas práticas)^(5,27)^.

Perspectivas futuras incluem desenvolvimento de indicadores de impacto que mensurem efetividade da formação em mudanças climáticas na prática profissional, bem como estabelecimento de redes colaborativas entre instituições de ensino para compartilhamento de experiências e recursos^(7,26)^. A consolidação dessa proposta curricular contribuirá para posicionamento da enfermagem brasileira como protagonista na resposta aos desafios climáticos, fortalecendo o SUS e promovendo equidade em saúde em âmbito nacional e internacional^(19,25)^.

Estudos recentes demonstram que a educação sobre clima e saúde impacta positivamente o conhecimento, a confiança e a intenção de agir dos estudantes de enfermagem^(28)^. Associações profissionais internacionais têm desenvolvido recomendações específicas para integração de estratégias de mitigação climática na prática de enfermagem^(5,29)^, demonstrando que a formação proposta se alinha às tendências globais da profissão. A literatura sobre desastres naturais evidencia desafios específicos nas condições de trabalho de enfermeiros^(9)^, como demonstrado em investigação recente sobre as condições laborais após terremoto na Turquia^(30)^, sublinhando a importância de preparação adequada durante a graduação.

### Limitações do estudo

Reconhece-se que este estudo, realizado no campo específico da enfermagem, apresenta limitações quanto à abordagem interdisciplinar essencial para a educação climática e limitações metodológicas quanto à intersubjetividade freireana, que exige diálogo direto entre os sujeitos na construção do conhecimento. A análise documental limitada pode não capturar plenamente perspectivas de outras áreas como ciências ambientais, políticas públicas e saúde coletiva. Como tal, apresenta limites devido à delimitação temporal, à natureza interpretativa da análise e ao dinamismo das políticas e diretrizes, que podem ser atualizadas após a coleta.

### Contribuições para a área da enfermagem, saúde ou políticas públicas

Este estudo contribui para debate sobre um desenho pedagógico específico para integração de mudanças climáticas na formação em enfermagem, e oferece fundamentação teórico-conceitual que articula determinantes socioambientais da saúde com formação profissional crítica, capacitando futuros enfermeiros para atuação territorializada. Nesse sentido, no âmbito clínico-assistencial, a proposta qualifica o raciocínio clínico e a tomada de decisão do enfermeiro ao incorporar e contribuir para identificar, analisar e intervir de forma efetiva diante das vulnerabilidades socioambientais. A formação proposta aponta práticas para justiça climática, equidade em saúde e resposta a emergências ambientais, fortalecendo a capacidade de *advocacy* e liderança dos profissionais na defesa do SUS e na promoção da saúde planetária.

Para a educação os eixos pedagógicos, que integram de maneira transversal os temas ambientais, sociais e de justiça climática ao currículo de enfermagem, orientam o desenvolvimento de práticas educativas críticas, contextualizadas e alinhadas aos ODS. O estudo identifica lacunas e silêncios nas políticas institucionais de formação em enfermagem no que se refere à educação climática e saúde planetária, propondo um referencial analítico que pode orientar futuras pesquisas sobre a efetividade dos currículos, o impacto das práticas pedagógicas na formação crítica de enfermeiros e a validação dos eixos propostos.

## CONSIDERAÇÕES FINAIS

Este estudo identificou marcos históricos e conceituais fundamentais que sustentam a integração das mudanças climáticas na formação em enfermagem, estruturando cinco eixos pedagógicos específicos. Os conceitos-chave identificados - justiça climática, saúde planetária, equidade, sustentabilidade e resiliência - posicionam a enfermagem como profissão comprometida com transformação social e ambiental.

O alinhamento evidenciado entre os eixos pedagógicos e os ODS 3, 4 e 13, bem como com as DCN/Enf, demonstra que a proposta fortalece a formação integral do enfermeiro. A operacionalização, através de metodologias ativas, simulação realística e projetos comunitários, mediada pelos princípios freireanos de dialogicidade e práxis, capacita futuros profissionais para atuação crítica e equitativa junto à populações vulnerabilizadas. A articulação entre ensino, serviço e comunidade promove formação contextualizada que valoriza saberes populares e práticas territoriais de resistência.

A proposta de eixos pedagógicos é fundamentada na premissa freireana de que a educação deve questionar estruturas opressoras, capacitando enfermeiros para advocacia profissional e liderança em justiça climática. Essa formação transcende preparação técnica para impactos climáticos, desenvolvendo consciência crítica sobre determinantes estruturais das vulnerabilidades socioambientais e capacidade de ação transformadora em territórios historicamente marginalizados. Deste modo, sugerem-se pesquisas que investiguem experiências práticas de implementação curricular, e percepções de estudantes, docentes e profissionais sobre a integração proposta e validação empírica dos eixos pedagógicos propostos.

## Data Availability

Os dados de pesquisa não estão disponíveis.
